# Insulin-Like Growth Factor 2 mRNA-Binding Protein 3 Influences Sensitivity to Anti-IGF System Agents Through the Translational Regulation of *IGF1R*

**DOI:** 10.3389/fendo.2018.00178

**Published:** 2018-04-20

**Authors:** Caterina Mancarella, Michela Pasello, Maria Cristina Manara, Lisa Toracchio, Evelina Fiorenza Sciandra, Piero Picci, Katia Scotlandi

**Affiliations:** ^1^CRS Development of Biomolecular Therapies, Experimental Oncology Laboratory, Orthopedic Rizzoli Institute, Bologna, Italy; ^2^Pathology Section, Orthopedic Rizzoli Institute, Bologna, Italy

**Keywords:** insulin-like growth factor 2 mRNA-binding protein 3, IGF1R, anti-IGF agents, Ewing sarcoma, personalized treatment

## Abstract

Insulin-like growth factor 2 (IGF2) mRNA-binding protein 3 (IGF2BP3) is an oncofetal protein that binds RNA, thereby influencing the fate of target transcripts. IGF2BP3 is synthesized *de novo* in cancer, where it promotes proliferation, drug resistance, and metastasis *via* both IGF2-dependent and IGF2-independent mechanisms. Ewing sarcoma (ES) is a rare bone and soft tissue tumor in which the IGF system plays a pivotal role. This study aimed to investigate the effect of IGF2BP3 on the regulation of the IGF system in ES. Among the components of the IGF axis, a direct significant correlation was identified between IGF2BP3 and IGF1R at mRNA and protein levels in two independent series of clinical specimens from patients with localized ES. After the formal demonstration of a direct association between IGF2BP3 and *IGF1R* mRNA using ribo-immunoprecipitation assay, we performed *in vitro* studies using A673 and TC-71 ES cell lines to demonstrate that IGF2BP3 loss promotes the downregulation of IGF1R and a decreased biological response to IGF1, represented by reduced migration and cell growth. Additionally, the compensatory activation of insulin receptor (IR) and its mitogenic ligand IGF2 is triggered in some but not all cell lines in response to IGF2BP3-mediated IGF1R loss. These findings have therapeutic implications because cells with a decreased expression of IGF2BP3/IGF1R axis but an increased expression of the IR/IGF2 loop display higher sensitivity to the dual inhibitor OSI-906 than do control cells. Therefore, studies on IGF2BP3, which was confirmed as a post-transcriptional regulator of IGF1R, provide a step forward in the identification of new mechanisms regulating the IGF system. In addition, our results demonstrate that the detection of IGF2BP3 expression should be combined with the assessment of the IGF1R/IR ratio to predict cell responses to anti-IGF1R/IR agents.

## Introduction

Insulin-like growth factor 2 (IGF2) mRNA-binding proteins (IGF2BPs) represent a family of oncofetal RNA-binding proteins (RBPs), including the paralogs IGF2BP1, 2, and 3, which control the localization, translation, and stability of mRNAs (1). IGF2BPs are mainly expressed during embryogenesis and absent in adult tissues (2, 3), but *in vitro* studies have demonstrated that IGF2BP1 and 3 are synthesized *de novo* in cancer, where they act as oncogenes promoting malignant processes including cell polarization, migration, morphological determination, proliferation, differentiation, and drug sensitivity (4–8). Structurally, the three proteins share 56% amino acid sequence homology and are characterized by two RNA-recognition motifs in the N-terminal region and four hnRNP-K homology domains in the C-terminal region, which are particularly involved in the binding, at 5′- or 3′-untranslated regions, of targets RNAs (9). IGF2BPs are located in the cytoplasm, mainly around the nucleus and in cell protrusions, as ribonucleoprotein granules acting as “safe-houses” for transcript targets (10). The comprehensive identification of IGF2BP targets is still under investigation, but the network of RNAs whose stability is regulated by each IGF2BP may predict the phenotype of IGF2BP-expressing cells and determine the involvement of these proteins in various crucial cellular functions [reviewed in Ref. (6, 11, 12)]. Each IGF2BP exhibits distinct RNA-binding properties and associates with variable targets, but all IGF2BPs are reported to associate with *IGF2* mRNA (2). IGF2, together with IGF1 and insulin, represents extracellular ligand belonging to the IGF system that also includes three receptors [insulin receptor (IR), IGF1R, and mannose 6-phosphate receptor (M6P/IGF2R)], and six known types of circulating IGF-binding proteins (13). Upon ligand binding, the kinase domains of the receptors are activated, and phosphorylation cascades are initiated through the PI3K (14) or MAPK (15) pathway with an impact on vital processes under physiological as well as pathological conditions including cancer. In tumor cells, the IGF system drives cell proliferation, differentiation, and survival during anoikis and after conventional and targeted therapies [for reviews, see Ref. (16, 17)]. Thus, IGF signaling, particularly involving IGF1R, has become an attractive target for the development of novel anticancer agents.

Among the IGF2BPs, IGF2BP3 has been particularly described to favor *IGF2* translation, thereby activating IGF signaling and promoting cell growth, proliferation, and resistance to ionic irradiation in different tumor types (18–20). Therefore, in this study, we investigated the value of IGF2BP3 as a novel regulator of the IGF system in Ewing sarcoma (ES), the second most frequent primary tumor of the bone affecting children and adolescents. The clinical history of ES remains disappointing because, despite an overall cure of approximately 70% in cases with localized disease, survival is lower than 30% in patients with metastatic disease. In addition, the reduction of severe treatment-related side effects still represents an urgent clinical need (21, 22). However, the discovery of novel treatment opportunities may not be imminent because ES is characterized by a recurrent *EWS/ETS* chromosomal translocation (*EWS-FLI1* in most of the cases) but otherwise an overall stable genome (23), leading to a general paucity of druggable mutations. The IGF system is a key participant in ES development and progression (24–28), and the functions of this system are mainly sustained by the autocrine production of IGF1, which induces constitutive IGF1R activation. The clinical use of agents blocking this axis has demonstrated remarkable efficacy in approximately 15% of ES patients (29–31) and has highlighted the need of more profound knowledge of this system to identify biomarkers of response. In the present study, we show that IGF2BP3 directly favors *IGF1R*, but not *IGF2*, translation in ES, supporting cell growth and migration in response to IGF1 and that different levels of IGF2BP3 expression can impact ES response to anti-IGF1R/IR agents.

## Materials and Methods

### Drugs

OSI-906 (linsitinib) was purchased from Selleckchem (Houston, TX, USA). IGF1 was purchased from Upstate (Waltham, MA, USA). Working dilutions of all drugs were prepared immediately before use.

### Cell Lines

Human ES cells TC-71 were kindly provided by T.J. Triche (Children’s Hospital, Los Angeles, CA, USA), A673 was provided by American Type Culture Collection (Rockville, MD, USA). Cell lines were authenticated by DNA fingerprinting (last control December 2017) and found mycoplasma-free by the MycoAlert mycoplasma detection kit (Lonza, Basel, Switzerland) (verified every 3 months). A673 and TC-71 cells were stably silenced for IGF2BP3 after transfection with the pLKO.1 vector containing IGF2BP3-specific short hairpin RNA (TRCN0000074673) and selection with puromycin 2 µg/ml (Sigma, St. Louis, MO, USA). Transfections were performed using TransIT-X2 (Mirus, Madison, WI, USA) according to the manufacturer’s protocol. The ES cell lines were grown as previously described (32).

### Patient Selection

Patients with localized ES who were enrolled in prospective studies and treated at the Rizzoli Institute were included in the present analysis (33). All patients had a diagnosis of ES made on representative specimens from open or needle biopsies based on histological, cytological, immunohistochemical features as well as the molecular presence of the chimeric product derived from ES-specific chromosomal translocations (34). Local treatment, performed after induction chemotherapy, consisted of radiation therapy, surgery or surgery followed by radiation therapy. In patients locally treated by surgery, histologic response to chemotherapy was evaluated according to the method proposed by Picci et al. (35). The patients’ clinical characteristics are summarized in Table 1. Clinical and follow-up data were updated to June 2014. The ethical committee of the Rizzoli Institute approved the study (0041040/2015), and informed consent was obtained.

**Table 1:** Clinico-pathological features of ES patients included in the study.

	qRT-PCR (*N* = 89)		IHC (*N* = 102)	
Characteristics	No	%	No	%
**Gender**				
Female	28	31.5	37	36.3
Male	61	68.5	65	63.7
**Age**				
≤14 years	34	38.2	46	45.1
>14 years	55	61.8	56	54.9
**Location**				
Extremity	68	76.4	93	91.2
Central	6	6.7	9	8.8
Pelvis	15	16.9	0	–
**Surgery**				
Yes	80	89.9	90	88.2
No	9	10.1	12	11.8
**Local treatment**				
RxT	9	10.1	12	11.8
RxT + surgery	17	19.1	27	26.5
Surgery	63	70.8	63	61.8
**Response to chemotherapy^a^**				
Good	32	40	52	59.8
Poor	48	60	35	40.2

*^a^Data available for 80 cases in qRT-PCR; data available for 87 cases in IHC*.

*ES, Ewing sarcoma; RxT, radiotherapy; IHC, immunohistochemistry; qRT-PCR, quantitative real-time PCR*.

### Gene Expression Analysis

Total RNA from snap-frozen tissue samples and/or cell lines was isolated using TRIzol Reagent (Invitrogen, Carlsbad, CA, USA). RNA quality and quantity were assessed by NanoDrop analysis (NanoDrop ND1000, ThermoFisher Scientific, Waltham, MA, USA) and/or by electrophoresis. To determine whether the extracted RNA was representative of ES, matching tissues were morphologically analyzed after hematoxylin–eosin staining. Oligo dT primers (Applied Biosystems, Foster City, CA, USA) were used to reverse transcribe RNA. Predesigned TaqMan probes (Applied Biosystem) for target genes were used to determine the expression level of the corresponding genes by quantitative real-time PCR (qRT-PCR) using a ViiA™ seven Real-Time PCR System (Applied Biosystems) according to the manufacturer’s instructions. The probes were as follows: *IGF2BP3* (Hs00559907_g1), *IGF1R* (Hs00181385_m1), and *ABCG2* (Hs00184979_m1). Primer sets for *IGF1* and *IGF2* were designed for SYBR Green-mediated quantification as previously reported (36). The Primer Express software (Applied Biosystems) was used to design appropriate primer pairs for the reference gene [glyceraldehyde-3-phosphate dehydrogenase (*GAPDH*)] as previously reported (36). Total RNA from primary cultures of human mesenchymal stem cells (hMSCs) obtained during surgery was also extracted and used as a calibrator. Two biological replicates were performed. In each replicate, samples were run in triplicate. Relative quantification analysis was performed using the 2^−ΔΔCt^ method (37).

### Immunohistochemistry (IHC)

Representative samples of paraffin-embedded ES tumors, at least three cores per patient, were included in a tissue microarray (TMA) and processed for IHC as previously described (38). The TMA was then incubated overnight at 4°C with the following primary antibodies: anti-IGF1Rβ antibody (Santa Cruz Biotechnology, Dallas, TX, USA) diluted 1:50, as previously reported (38), or anti-IGF2BP3 antibody (Santa Cruz Biotechnology) diluted 1:50. The samples were classified based on positivity scores as follows: “low expression” (categorized as 0 for statistical analysis), when no staining or low positivity was observed (intensity of the staining was scored as +/– or + ––), or “high expression” (categorized as 1 for statistical analysis), when a diffused immunostaining was present (intensity of the staining was scored as + + − or + + +).

### Western Blotting

Cell lysates were prepared and processed as previously described (32). Membranes were incubated overnight with the following primary antibodies: anti-IRβ, anti-IGF2BP3, anti-GAPDH, anti-IGF1Rβ, anti-Lamin B (Santa Cruz Biotechnology), anti-phospho-ERK 1/2, anti-ERK 1/2, anti-phospho-Akt (Ser473), and anti-Akt (Cell Signaling Technology, Beverly, MA, USA) antibodies. Anti-rabbit, anti-mouse (GE Healthcare, Little Chalfont, UK), and anti-goat (Santa Cruz Biotechnology) antibodies conjugated to horseradish peroxidase were used as secondary antibodies.

### *In Vitro* Assays

Sensitivity to OSI-906 was assessed after 72 h of treatment with the TACS^®^ MTT Cell Proliferation Assay kit (Trevigen, Inc., Gaithersburg, MD, USA) according to the manufacturer’s instructions. Cells were seeded into 96-well plates (2,500 cells/well) in IMDM plus 10% FBS. After 24 h, various concentrations of OSI-906 (0.3–10 µM) were added. Motility assay was performed using Transwell chambers (CoStar, Cambridge, MA, USA). 10^5^ cells in IMDM plus 1% FBS were seeded in the upper compartment, and IMDM plus 1% FBS and IGF1 (50 ng/ml) was added to the lower compartment of the chambers. To assess the response to IGF1, 5 × 10^4^ cells were seeded in 24-well plates, serum starved for 24 h and treated with IGF1 (50 ng/ml) for 24 h. Trypan Blue cell count was employed to assess the response to IGF1. For each independent experiment, samples were in duplicate (Trypan Blue) or triplicate (MTT assay; Motility assay).

### Ribo-Immunoprecipitation (RIP) Assay

For RIP assays, cells plated in 100-mm Ø dishes, to generate 4–6 mg of total protein, were washed twice with PBS, harvested, and extracted for 30 min on ice in 250 µl of ice-cold lysis buffer [100 mM KCl, 5 mM MgCl_2_, 10 mM HEPES (pH 7.0), 0.5% Nonidet P-40, and 1 mM dithiothreitol (DTT)] supplemented with RNase and protease inhibitors. Extracts were cleared by centrifugation at 4°C for 15 min at 12,000 rpm. Supernatants were collected and diluted in NT-2 buffer [150 mM NaCl, 1 mM MgCl_2_, 50 mM Tris–HCl (pH 7.4), and 0.05% Nonidet P-40] supplemented with protease and RNase inhibitors and 1 mM DTT. To preclear the cytoplasmic extracts, 50 µl of a suspension of Protein G plus/Protein A agarose beads (Merck Millipore) was added to the supernatant for 1 h at 4°C. For immunoprecipitation, precleared extracts were incubated with 150 µl of Protein G plus/Protein A agarose beads precoated with the same amount of either nonimmune mouse IgG (Santa Cruz Biotechnology) or anti-IGF2BP3 antibody (10 µg) in 800 µl of NT-2 buffer [150 mM NaCl, 1 mM MgCl_2_, 50 mM Tris–HCl (pH 7.4), and 0.05% Nonidet P-40] containing 1 mM DTT, RNase inhibitor, and protease inhibitors for 4 h at 4°C with rotation. The beads were washed 10 times with ice-cold NT-2 buffer. RNA was extracted by adding TRIzol directly to the beads, and qRT-PCR was performed on equivalent amounts of samples to quantify protein-bound mRNAs.

### Statistical Analysis

Correlations between gene expression levels were assessed using Spearman’s rank test while association between protein expression levels was assessed using Fisher’s exact test. All *p*-values were two-sided. *p*-Value <0.05 was considered statistically significant. Differences among means were analyzed using Student’s *t*-test or one-way ANOVA when experimental data included more than two groups.

## Results

### IGF2BP3 Expression Is Correlated With IGF1R Expression in Primary ES Tumors

**Figure 1:**
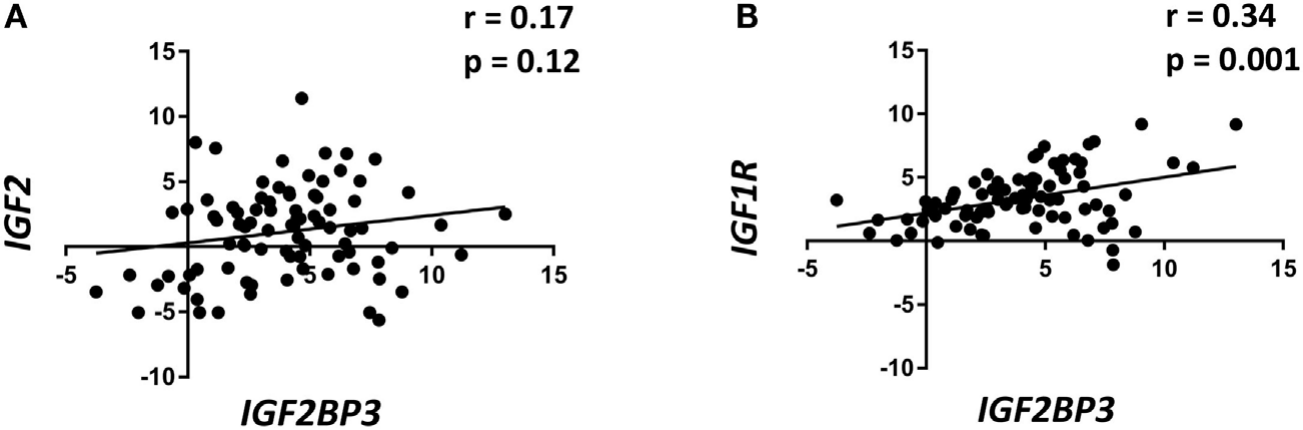
Insulin-like growth factor 2 (IGF2) mRNA-binding protein 3 (IGF2BP3) expression is directly correlated with IGF1R expression in Ewing sarcoma (ES) cases. Scatter plot displaying the correlation between *IGF2BP3* and *IGF2* **(A)** or *IGF1R* **(B)** mRNA levels in 89 ES cases. Correlation coefficient (*r*) and *p*-value were calculated using Spearman’s correlation test. Human mesenchymal stem cells were used as calibrators (2^−ΔΔCt^ = 1).

Data from the literature indicate that IGF2BP3 directly regulates *IGF2*, thereby enhancing IGF1R activity. Therefore, the expression of *IGF1R* and *IGF2* were evaluated using qRT-PCR in a retrospective series of 89 primary ES specimens and compared with the expression of *IGF2BP3* (Table 1). *IGF2BP3* expression was not correlated with *IGF2* expression (Figure 1A), but a direct correlation was found between *IGF1R* and *IGF2BP3* expression (Figure 1B). This association was confirmed at protein level. We analyzed the protein expression of IGF2BP3 and IGF1R in an independent series of 102 ES patients (Table 1). As observed at the mRNA level, IGF1R protein expression was found to be significantly associated with IGF2BP3 expression [IGF1R: low expression 29/102, high expression 73/102; IGF2BP3: low expression 62/102, high expression 40/102; IGF1R and IGF2BP3 protein levels in agreement (either low expression and high expression) in 61/102 cases; *p* = 0.001, Fisher’s exact test], further verifying the association between the two molecules.

### IGF2BP3 Binds to and Stabilizes *IGF1R* mRNA and Positively Modulates Its Functions

**Figure 2:**
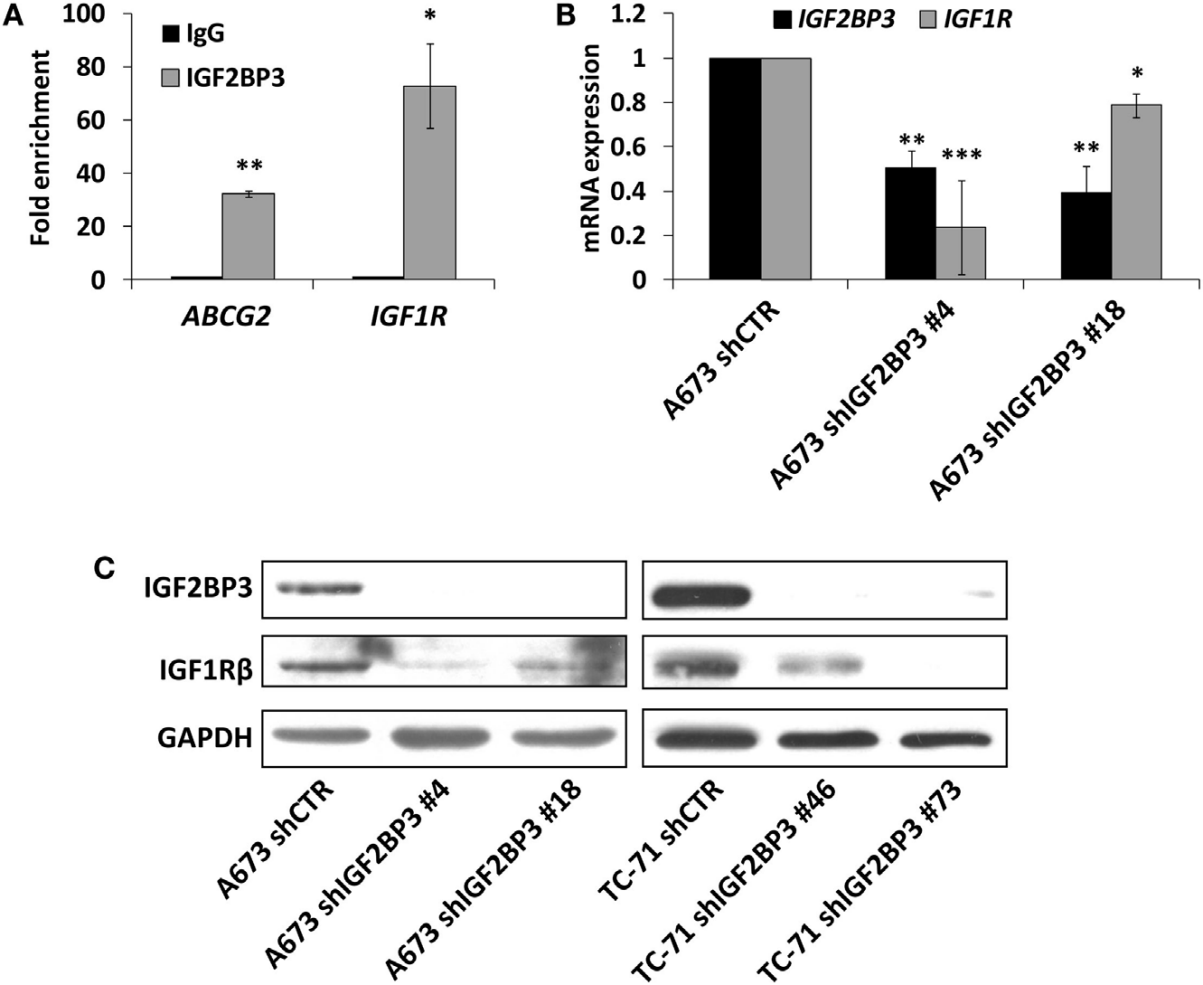
Assessment of *IGF1R* as an insulin-like growth factor 2 mRNA-binding protein 3 (IGF2BP3) mRNA target in Ewing sarcoma (ES) cells. **(A)** Quantitative real-time PCR analysis of IGF2BP3-associated mRNAs isolated from the cytoplasmic extracts of A673 cells by immunoprecipitation using an anti-IGF2BP3 antibody. Non-immune goat IgG was used as a negative control. *ABCG2* was used as positive control. Columns represent the mean values of two independent experiments, in which samples were run in triplicates, and the bars represent the SE. *p < 0.05, **p < 0.01, Student’s *t*-test. **(B)** mRNA expression levels of *IGF2BP3* and *IGF1R* in IGF2BP3-depleted or empty vector-transfected (shCTR) A673 ES cells. *GAPDH* was used as a housekeeping gene. Columns represent the mean values of at least two independent experiments, in which samples were run in triplicates, and the bars represent the SE. *p < 0.05, **p < 0.01, ***p < 0.001, one-way ANOVA with respect to shCTR. **(C)** Western blotting showing IGF2BP3 and IGF1R expression in IGF2BP3-depleted or empty vector-transfected (shCTR) A673 (left) or TC-71 (right) ES cells. GAPDH was used for normalization. Data from one experiment representative of three.

**Figure 3:**
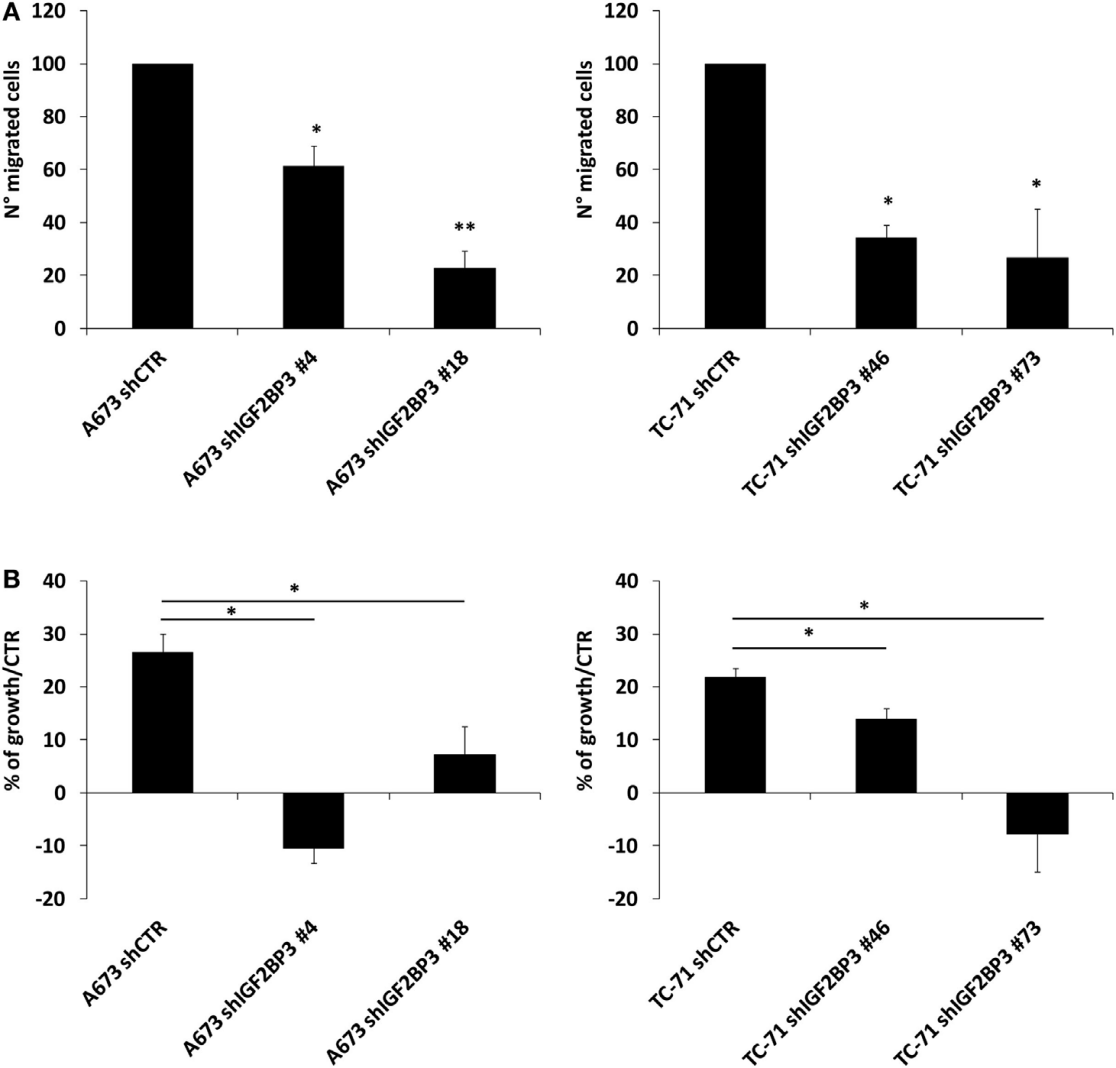
Evaluation of the oncogenic potential of insulin-like growth factor 2 mRNA-binding protein 3 (IGF2BP3)/IGF1R in Ewing sarcoma (ES) cell lines. **(A)** Migration of IGF2BP3-depleted or empty vector-transfected (shCTR) A673 or TC-71 ES cells in response to IGF1 (50 ng/ml) stimulation. Columns represent the mean values of two independent experiments, in which samples were run in triplicate, and the bars represent the SE. **(B)** Growth capacity of IGF2BP3-depleted or empty vector-transfected (shCTR) A673 or TC-71 ES cells after 24 h of IGF1 (50 ng/ml) stimulation. Trypan blue staining was used for cell counting. Columns represent the mean values of two independent experiments, in which samples were run in duplicates, and the bars represent the SE. **(A,B)** *p < 0.05, **p < 0.01, one-way ANOVA.

The interaction between IGF2BP3 and *IGF1R* mRNA was validated *in vitro* by RIP assay using anti-IGF2BP3 and control IgG antibodies; after extracting RNA from immunoprecipitated samples, qRT-PCR analysis was used to measure *IGF1R* levels in each sample. *IGF1R* was highly enriched in IGF2BP3 immunoprecipitated samples relative to that in IgG immunoprecipitated samples, revealing that IGF2BP3 selectively associates with *IGF1R* (Figure 2A). To investigate the biological significance of IGF2BP3 binding to *IGF1R*, we first examined whether IGF2BP3 regulates *IGF1R* stability. Depletion of IGF2BP3 in ES cells significantly reduced *IGF1R* mRNA (Figure 2B) and protein levels (Figure 2C). Compared with control cells, IGF2BP3-depleted cells displayed reduced migration (Figure 3A) and growth (Figure 3B) in response to IGF1, confirming that IGF2BP3 levels substantially impact IGF1R-mediated functions in ES cells.

### Expression of IGF2BP3 May Indirectly Regulate the IR/IGF2 Loop and Sensitivity to Anti-IGF1R/IR Agents

**Figure 4:**
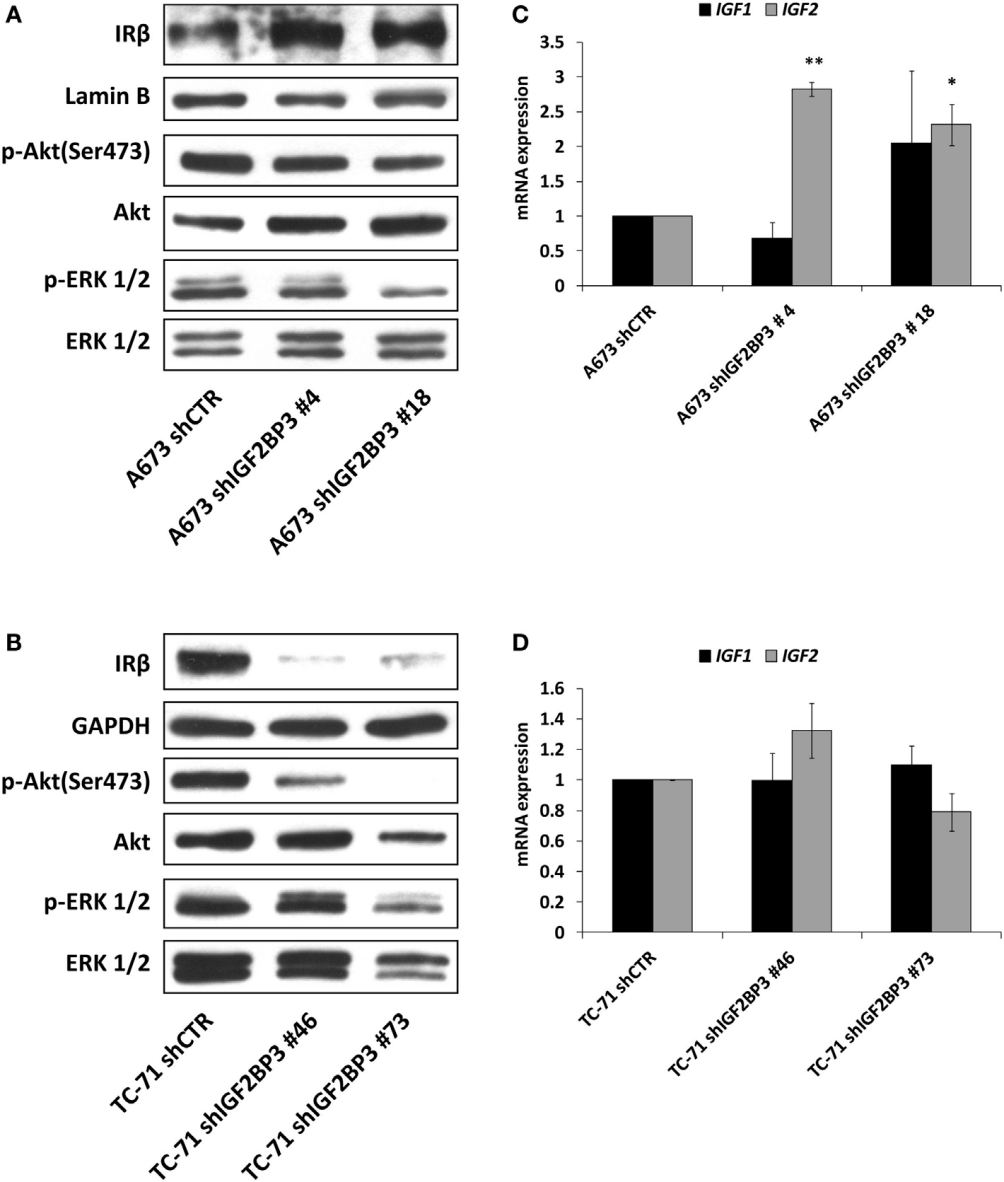
Insulin receptor (IR) signaling in response to insulin-like growth factor 2 (IGF2) mRNA-binding protein 3 (IGF2BP3)-mediated IGF1R regulation. **(A,B)** Western blotting depicting the expression of IR and phosphorylation of Akt and ERK 1/2 in IGF2BP3-depleted or empty vector-transfected (shCTR) **(A)** A673 or **(B)** TC-71 Ewing sarcoma (ES) cells. GAPDH, Lamin B, and total proteins were used for normalization purposes. Data from one experiment representative of three. **(C,D)** mRNA level of *IGF1* and *IGF2*, expressed as 2^−ΔΔCt^, in IGF2BP3-depleted or empty vector-transfected (shCTR) **(C)** A673 or **(D)** TC-71 cells. Columns represent the mean values of at least two independent experiments, in which samples were run in triplicates, and the bars represent the SE. *p < 0.05, **p < 0.01, one-way ANOVA compared with shCTR.

It has been demonstrated that ES cells can rapidly counteract IGF1R blockade induced by selective anti-IGF1R antibodies by switching from the canonical IGF1/IGF1R pathway to the alternative IGF2/IR signaling pathway (36, 39). Thus, we postulated that a similar mechanism of resistance is triggered by IGF2BP3. Indeed, compared with control cells, in addition to exhibiting decreased expression of IGF1R, IGF2BP3-silenced A673 cells displayed increased levels of IR (Figure 4A) and *IGF2* (Figure 4C). Additionally, as a consequence of the activation of this compensatory loop, phosphorylation of downstream signaling components Akt and ERK 1/2 was maintained in IGF2BP3-silenced cells (Figure 4A). However, this was not a general phenomenon, because we were unable to observe the same alterations in the TC-71 experimental models in which IR was downregulated similarly to IGF1R (Figure 4B), and marked decreased level of Akt phosphorylation was observed (Figure 4B), but no modulation in the expression of the ligands was detected (Figure 4D) when the cells were depleted of IGF2BP3.

**Figure 5:**
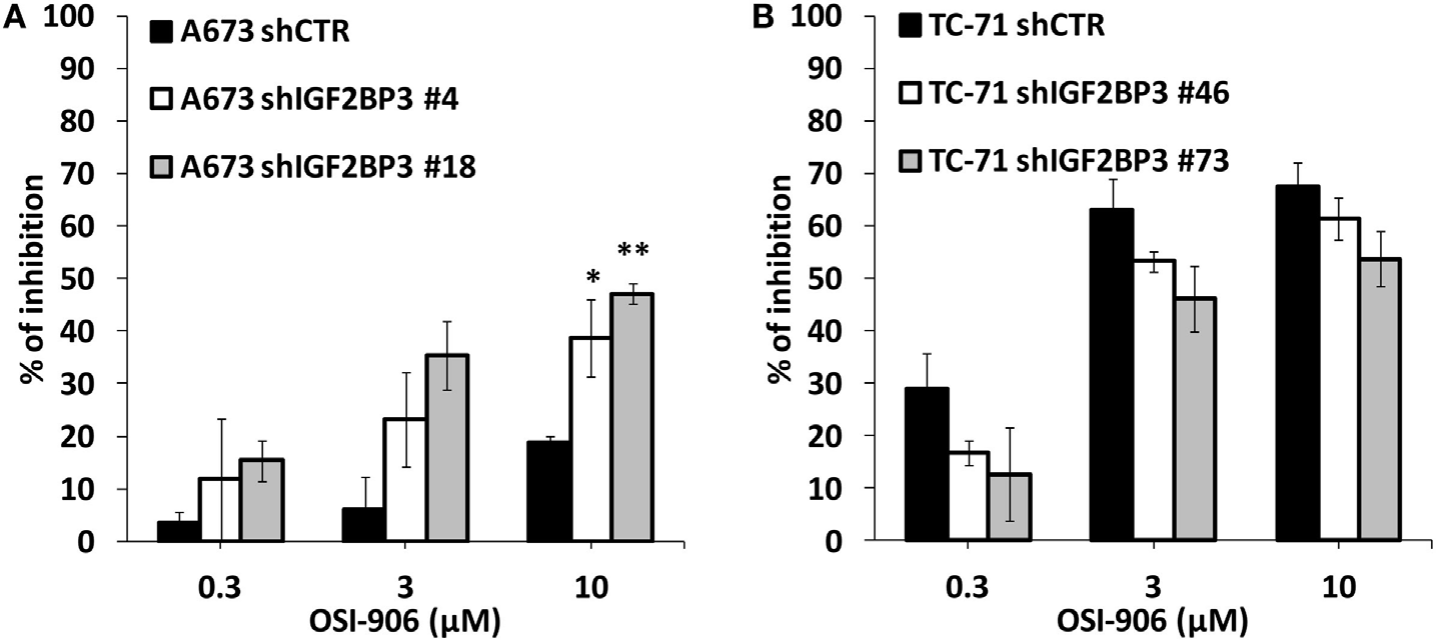
Efficacy of agents blocking the IGF system in insulin-like growth factor 2 mRNA-binding protein 3 (IGF2BP3)-depleted models. Cell growth was assessed using the MTT assay after 72 h of exposure to OSI-906 at indicated doses in IGF2BP3-depleted or empty vector-transfected (shCTR) A673 **(A)** and TC-71 **(B)** Ewing sarcoma cells. Results are displayed as the percentage of growth inhibition relative to that in untreated control cells. Histograms represent the mean of three independent experiments, in which each sample was in triplicate, and bars represent the SE. *p < 0.05, **p < 0.01, one-way ANOVA compared with shCTR.

The data presented above indicate that IGF2BP3 directly and indirectly affects the expression of IGF system components. Therefore, we tested the sensitivity of ES cells with differential expression of IGF2BP3 to anti-IGF agents. Based on the reported role of IGF1R as a major driver of cancer development and progression, several pharmacological inhibitors were developed including the small-molecule inhibitor OSI-906, which can block both IGF1R and IR. Accordingly, compared with control cells, IGF2BP3-depleted A673 cells, which showed decreased IGF1R but increased IR/IGF2 expression and active PI3K and MAPK signaling, displayed an increased sensitivity to the dual inhibitor OSI-906 (Figure 5A). However, this was not the case for IGF2BP3-depleted TC-71 cells, where no compensatory mechanism mediated by IR signaling was observed (Figure 5B).

## Discussion

The rationale for the development of agents against IGF1R was provided by experimental evidence showing that the receptor is necessary for malignant transformation, while low IGF activity was shown to protect against cancer (40). Despite promising preclinical results, the negative results obtained by phase 2/3 trials using selective anti-IGF1R antibodies have prompted the cessation of several pharma programs and blocked the research on the IGF system. However, even the failed studies included patients who received some benefit, especially ES patients. On one hand, this evidence confirmed the therapeutic importance of the IGF1R pathway, but on the other, it supported the need for deeper insights into the IGF system to help the identification of biomarkers of response [reviewed in Ref. (41)]. In this study, we demonstrate that IGF2BP3 is a novel regulator of *IGF1R* expression, with implications for both cellular malignancy and therapy. Direct activating mutations in IGF1R have not been described, and regulation of *IGF1R* gene expression is generally achieved at the level of transcription (42). In ES cells, EWS-FLI1, the oncogenic driver of this tumor (43), was found to directly bind to the *IGF1R* promoter and sustain IGF1R expression (44, 45). In this study, we provide evidence of an RBP-mediated post-transcriptional regulation of IGF1R, proposing the possibility of an additional regulatory mechanism that operates in ES cells. In contrast to other tumors in which the effect of IGF2BP3 on the IGF system is primarily mediated by *IGF2* mRNA regulation (4, 20), in ES, IGF2BP3 mainly affects *IGF1R*. The expression of IGF1R and IGF2BP3 are correlated in clinical ES samples, while in experimental models, although the small number of independent experiments suggests caution in the interpretation of the data, we demonstrated that IGF2BP3 can bind to and stabilize *IGF1R* mRNA, favoring the expression of the receptor and its functions. In fact, when IGF2BP3 is silenced, ES cells express low levels of IGF1R and respond less to the IGF1 stimulus in terms of cell growth and migration. No direct relationship was observed between IGF2BP3 and the ligands *IGF1* and *IGF2*. However, *IGF2*, together with IR, may be induced at least in some cell lines to activate an IR-mediated mitogenic pathway that can compensate for the IGF2BP3-mediated repression of IGF1R. Therefore, IGF2BP3 can directly or indirectly module the sensitivity of cells to anti-IGF1R/IR agents, and this molecule should thus be added to the list of proteins that are analyzed for personalized treatment with agents targeting the IGF system. This is highly required especially because clinical responses to anti-IGF1R is observed in approximately 15% in ES, the tumor type with the best response to this targeted approach, while clinical response was registered in only a small (1–5%) but distinct proportion of patients with solid tumors (46, 47). In thyroid tumors (20), the high expression of IGF2BP3 sustains IGF2 levels and leads to an increased sensitivity to OSI-906. In contrast, in ES, the low expression of IGF2BP3 inhibits IGF1R expression and indirectly induces IR/IGF2 activation, leading to an increased sensitivity to the dual inhibitor OSI-906. Considering that the upregulation of IR and IGF2 after IGF2BP3 depletion did not appear to be a general phenomenon, reflective of heterogeneity in tumor response, we strongly advocate for the need to evaluate the IGF1R/IR ratio together with the expression of IGF2BP3 to predict therapeutic response to agents targeting IGF1R and/or IR before and during any treatment.

Overall, our data indicate that IGF2BP3 represents a novel regulator of IGF1R that has a wide impact on the IGF system in ES cells. This interaction strongly affects response to IGF1, one of the major growth factors influencing ES malignancy. The results of this study provide early, preclinical evidence that the assessment of IGF2BP3 expression combined with the IGF1R/IR ratio may be used to predict therapeutic responses to the dual IGF1R/IR inhibitor OSI-906, providing new insights for a more precise and rational administration of anti-IGF system agents in ES.

## Ethics Statement

This study was carried out in accordance with the recommendations of the ethical committee of the Rizzoli Institute that approved the study (0041040/2015). All subjects gave written informed consent.

## Author Contributions

CM and KS conception and design of the study. CM, MP, MCM, EFS, and LT acquisition of data. CM, MP, PP, and KS analysis and interpretation of data. CM, MP, and KS drafting or revising the work. All the authors read and approved the final manuscript.

## Conflict of Interest Statement

The authors declare that the research was conducted in the absence of any commercial or financial relationships that could be construed as a potential conflict of interest.
